# Layers of Hope: How Graphene and Nanostructures Hold Promise for Cancer Therapy

**DOI:** 10.3390/ijms27052336

**Published:** 2026-03-02

**Authors:** Beatriz Fumelli Monti Ribeiro, Gláucia Maria Machado-Santelli

**Affiliations:** Postgraduate Program in Systems Biology, Institute of Biomedical Sciences, University of Sao Paulo (USP), Sao Paulo 05508900, Brazil; beatrizfumelli@usp.br

**Keywords:** cancer, nanomedicine, targeted therapies and nano-cell interactions

## Abstract

Cancer remains a challenge in modern medicine, characterized by high mortality rates and significant variability in treatment response. The urgent need for more effective and targeted therapies has driven the exploration of innovative strategies, including nanomedicine, which promises precise therapeutic delivery to tumor cells and minimal off-target effects in healthy tissues. This review critically summarizes recent advances in the use of nanomaterials for cancer therapy, focusing on graphene-based materials and dendrimers, whose complementary physicochemical architectures enable the rational design of hybrid nanoplatforms with enhanced therapeutic efficacy and biological performance. Due to their distinctive properties, such as high surface area, tunable architecture, and versatile surface functionalization, nanomaterials have emerged not only as drug delivery platform but also as active modulators of cancer-associated cellular processes. We discuss current evidence on nano-cell interactions, elucidating how these interactions can influence key cellular pathways relevant to tumor progression and treatment response. Despite their promise, a comprehensive understanding of the interactions between these nanomaterials and human cells is critical for clinical translation. A deeper mechanistic understanding of these interactions is essential to guide potential pathways for the development of effective therapeutics, paving the way for future advances in nanomedicine.

## 1. The Emperor of All Maladies

Cancer remains one of the primary causes of mortality worldwide, accounting for one in every six deaths [1]. It is estimated that one in five individuals will develop cancer during their lifetime [2].

This complex disease is characterized by the uncontrolled growth of abnormal cells that can invade nearby tissues and spread from the primary site to distant organs [3,4,5]. The ability to invade and metastasize develops as tumors acquire multiple malignant traits driven by genetic and epigenetic changes that disrupt key cellular pathways, including DNA repair, cell cycle regulation, and immune surveillance [6,7].

**Figure 1:**
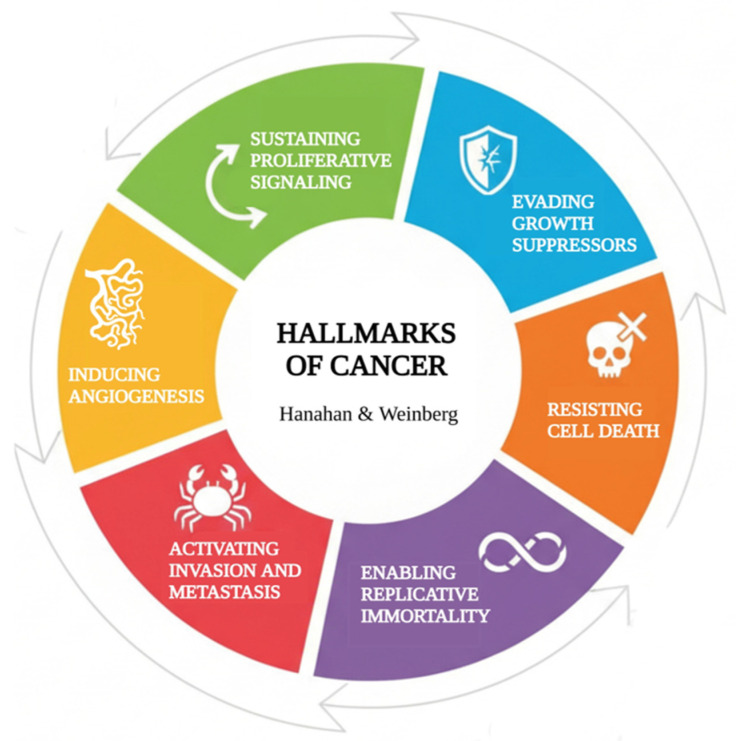
Key features of cancer. Illustration created by Gemini and adapted with BioRender, based on Hanahan and Weinberg [3,4].

Over the years, several additional mechanisms have been incorporated into the conceptual framework of the hallmarks of cancer [8], such as metabolic reprogramming, immune evasion, genomic instability, and tumor-associated inflammation. However, this review focuses on the six classical hallmarks originally proposed by Hanahan and Weinberg [3,4]: sustaining proliferative signaling, evading growth suppressors, resisting cell death, enabling replicative immortality, inducing angiogenesis, and activating invasion and metastasis (Figure 1). These hallmarks form the conceptual foundation of tumor biology and remain central to understanding the progression of cancer.

Highlighting these aspects is particularly relevant in the context of this review, which examines how interactions between nanomaterials and cancer cells influence key biological processes associated with tumor progression and therapy resistance (Table 1). Elucidating these nano-bio interactions is critical for the development of nanoscale therapeutic strategies capable of addressing the multifactorial nature of cancer.

**Figure 2:**
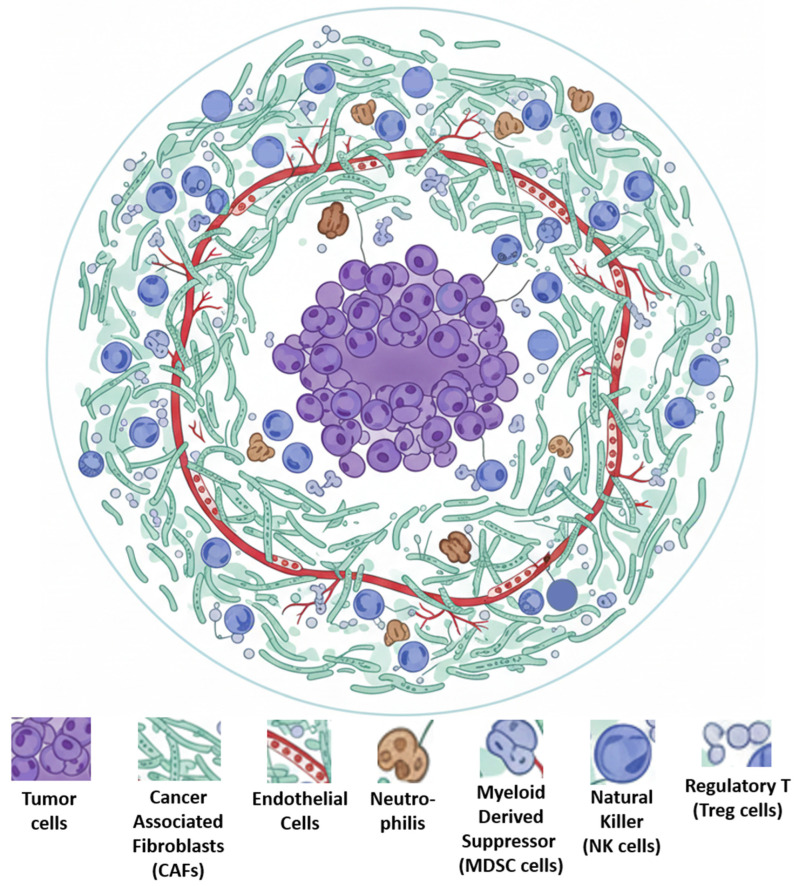
Cells of the tumor microenvironment. The tumor microenvironment is dynamic, undergoing changes in cellular composition and extracellular matrix organization throughout tumor progression, which supports primary, invasive, and metastatic growth. Image generated with Gemini and modified using BioRender.

Moreover, tumors are not composed solely of transformed cells but also of a diverse repertoire of recruited host cells (Figure 2), including tumor cells, cancer-associated fibroblasts (CAFs), endothelial cells, and immune cell populations such as neutrophils, myeloid-derived suppressor cells (MDSCs), natural killer (NK) cells, and regulatory T cells (Tregs). These cells interact with malignant cells and contribute to tumor progression by modulating the tumor microenvironment [26,27].

Although the hallmarks of cancer offer a conceptual framework for malignant phenotypes, they do not explain the etiological and mechanistic processes through which these capabilities are acquired. The acquisition of hallmark features reflects the cumulative impact of genetic alterations and epigenetic reprogramming, shaped over time by environmental and lifestyle-related exposures. Understanding the factors that initiate and promote these changes is therefore essential for contextualizing cancer as a disease that emerges from long-term interactions between cells and their surroundings [28,29].

Cancer can be caused, either partially or entirely, by modifiable lifestyle factors such as tobacco and alcohol consumption, obesity, nutrient-poor diets, physical inactivity, and inadequate sun exposure. However, beyond these behavioral factors, a wide range of environmental and occupational exposures also contribute to carcinogenesis. Genotoxic substances present in the environment, including pesticides, asbestos, silica, benzene, and other chemical solvents, have been associated with an increased risk of several cancer types. In addition, biological agents such as oncogenic viruses (HPV, HBV, and EBV) play a significant role in tumor initiation and progression. The incidence of cancer also increases with age due to the progressive accumulation of DNA damage and the decline in the efficiency of cellular repair mechanisms [30,31,32,33].

Female breast cancer represents the most prevalent malignancy worldwide and the predominant cancer type among women, followed by lung cancer, the most prevalent malignancy among men [2].

### 1.1. Breast Cancer

**Figure 3:**
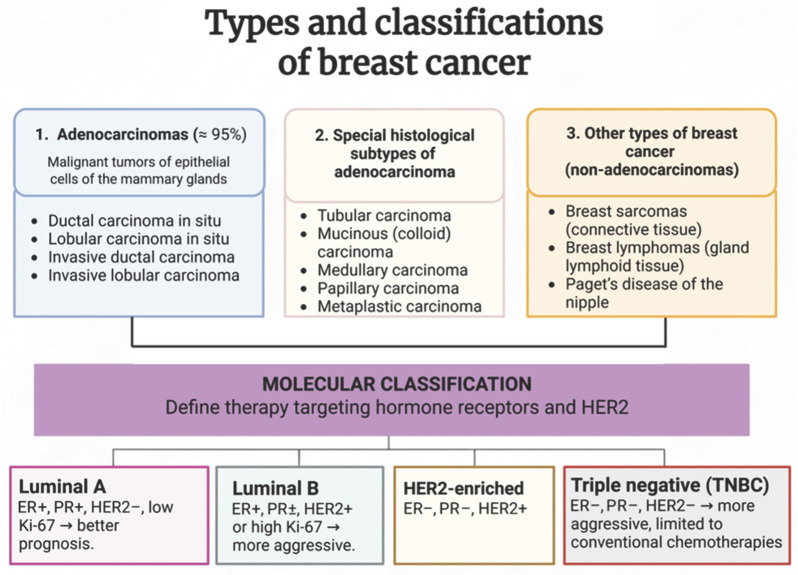
Types and classifications of breast cancer. ER: estrogen receptor (+positive/−negative), PR: progesterone receptor (+positive/−negative), HER2: Human Epidermal Growth Factor Receptor 2 (+positive/−negative). Image created with BioRender.

It is estimated that up to 10% of breast cancer cases are associated with hereditary genetic mutations and family history [34]. Depending on the site of origin, breast tumors are classified into distinct subtypes (Figure 3). Most breast tumors originate from the epithelial cells of the lactiferous ducts and are classified as ductal carcinomas. Tumors that arise from the cells of the mammary lobules (the milk-producing glands) are known as lobular carcinomas [35]. In addition to these more common types, less prevalent forms of breast cancer also exist, including Paget’s disease.

Triple-negative breast cancer (TNBC) represents a subtype characterized by the absence of estrogen (ER) and progesterone (PR) receptors, as well as the lack of overexpression or amplification of the human epidermal growth factor receptor 2 (HER2). Among breast cancer subtypes, luminal tumors are generally associated with a more favorable prognosis, with Luminal B tumors displaying higher proliferative activity and a more aggressive clinical behavior than Luminal A tumors. In contrast, the molecular profile of TNBC precludes the use of targeted therapies such as tamoxifen (a hormonal blocker) or trastuzumab (an anti-HER2 monoclonal antibody), thereby limiting treatment options primarily to conventional chemotherapy [36,37]. Although less common, TNBC is considered the most aggressive subtype due to its high recurrence rate, rapid progression, and poor clinical prognosis.

Accurately distinguishing among tumor types is essential for selecting the most appropriate therapeutic strategy [38].

### 1.2. Lung Cancer

**Figure 4:**
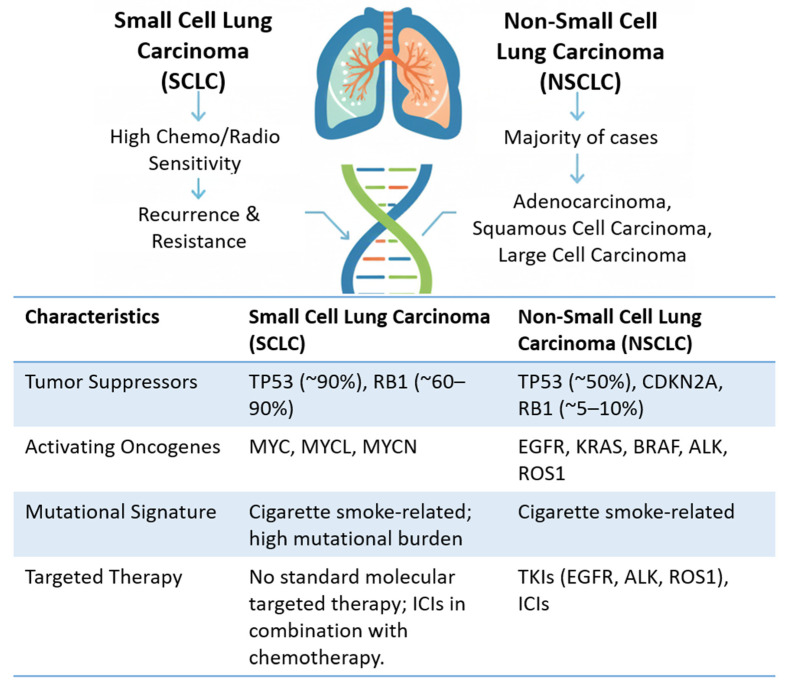
Overview of lung cancer. Key risk factors, recurrent genetic alterations, and biological consequences of SCLC and NSCLC are summarized. Image generated with Gemini.

Lung tumors are divided into two major groups with distinct therapeutic and prognostic strategies: small cell lung carcinoma (SCLC) and non-small cell lung carcinoma (NSCLC). SCLC is characterized by an initially high sensitivity to chemotherapy and radiotherapy, but with a strong tendency to recur and develop resistance. NSCLC represents the majority of cases and is subdivided into adenocarcinoma, squamous cell carcinoma, and large cell carcinoma [39]. Traditional classification relies primarily on morphology, immunohistochemistry, and histopathology, whereas advances in molecular profiling have revealed recurrent genetic alterations with diagnostic, prognostic, and therapeutic significance [40]. For example, adenocarcinomas frequently harbor activating mutations in EGFR, KRAS, and BRAF, as well as rearrangements in ALK and ROS1, whereas squamous cell carcinomas more commonly exhibit TP53, CDKN2A, and FGFR1 alterations. Such molecular characterization has led to the development of targeted therapies and immune checkpoint inhibitors (ICIs), transforming the management of NSCLC [41] (Figure 4).

The main risk factors for lung cancer remain smoking and passive smoke exposure, which contribute to a high mutational burden and characteristic mutation signatures. Additionally, occupational and environmental exposures (asbestos, silica, benzene, and other solvents), air pollution, biomass burning, and high-dose radiation increase risk [42]. Emerging evidence also highlights the influence of genetic predisposition, epigenetic modifications, and interactions between environmental and lifestyle factors in shaping tumor initiation and progression. These complex interactions underscore the heterogeneity of lung cancer and the importance of integrating molecular and environmental data to guide prevention, diagnosis, and treatment strategies.

The recognition of cancer as a disease characterized by interconnected and adaptive cellular characteristics highlights the need for approaches capable of capturing its biological complexity. In this context, novel therapeutic strategies and nanomaterials offer a valuable interface between physical sciences and tumor biology, providing tools to examine how cancer cells are involved with fundamental processes related to proliferation, survival, and invasion [43,44,45]. In the following sections, we discuss the advantages and limitations of these technologies.

## 2. Emerging Therapeutic Frontiers: Novel Strategies in Oncology

**Figure 5:**
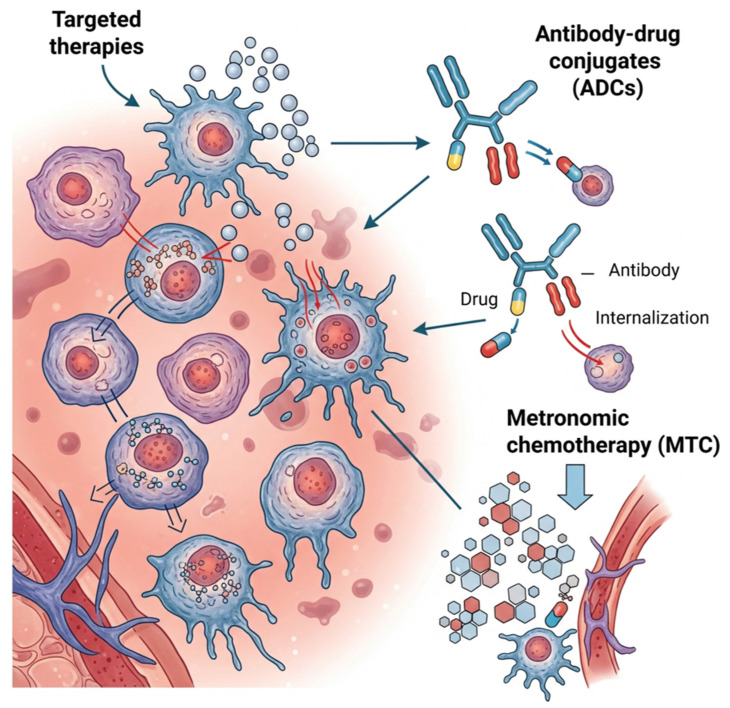
New therapeutic strategies in oncology: The image summarizes emerging fronts in cancer treatment, highlighting the role of targeted therapies, antibody-drug conjugates (ADCs), and metronomic chemotherapy as promising strategies to combat the disease with better specificity and lower toxicity. Image generated with Gemini and modified with BioRender.

New therapeutic strategies have been proposed for cancer treatment (Figure 5), including cell cycle modulation [46], interference with cellular metabolism [47], targeted therapies focusing on signaling pathways and molecular targets [48,49], the use of monoclonal antibodies [50,51,52], and applications of antibody-drug conjugates and immunotherapies [53,54,55,56,57].

Another recent strategy is metronomic chemotherapy (MTC), characterized by the continuous administration of reduced doses of chemotherapeutic agents at regular intervals, allowing effective drug levels with lower toxicity. One of the main effects attributed to this approach is its antiangiogenic action [58,59].

Antibody-drug conjugates (ADCs) represent a promising therapeutic strategy in cancer treatment, combining the specificity of monoclonal antibodies with potent cytotoxic agents [60,61,62]. These hybrid molecules are formed by covalently linking a tumor-specific antibody to a drug [63]. The mechanism of action of ADCs involves three main steps: binding of the antibody to the tumor antigen, endocytosis of the ADC-antigen complex, and subsequent release of the active drug following intracellular degradation [64,65]. Clinical efficacy depends on molecular design, antigen type, ADC stability in circulation, and the efficiency of drug release in the tumor microenvironment. Several ADCs have already been approved or are in clinical trial phases by the U.S. Food and Drug Administration (FDA), showing potential to reduce toxicity and improve antitumor response [66,67].

Although these novel therapeutic strategies have demonstrated improved specificity and efficacy, their clinical impact is often constrained by limited tumor penetration and systemic toxicity. In this context, nanoparticles can serve as “invisible soldiers,” capable of navigating biological barriers, enhancing drug accumulation in tumors, and offering multifunctional platforms that integrate therapy and imaging [68,69,70,71]. Due to their nanoscale dimensions and physical-chemical properties, these materials enable interactions with cellular components at size scales relevant to membranes, protein complexes, and intracellular structures. As a result, the interactions between nanomaterials and cells can be explored to investigate how pathways associated with distinctive features are organized and modified within cancer cells, establishing nanomaterials as experimental probes that extend and complement conventional biological models [72,73,74,75]. Below, we examine selected nanoparticle platforms, highlighting their mechanistic effects on cancer cells and their potential translational applications.

## 3. Subcellular Interventions: Nanomedicine as a Strategy Against Cancer

**Figure 6:**
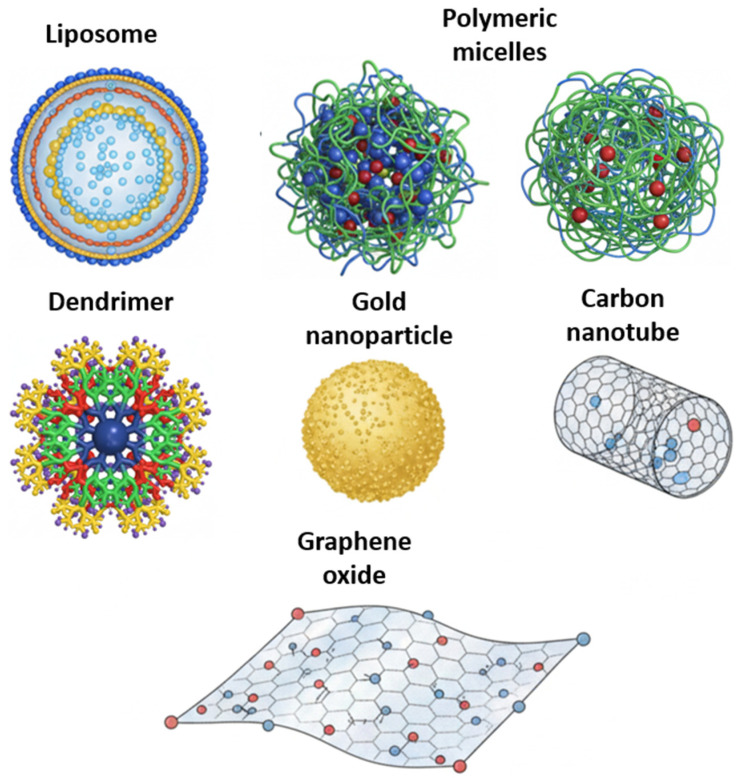
Schematic image of different nanomaterials that can be used as drug carriers. Image generated with Gemini and modified with BioRender.

**Figure 7:**
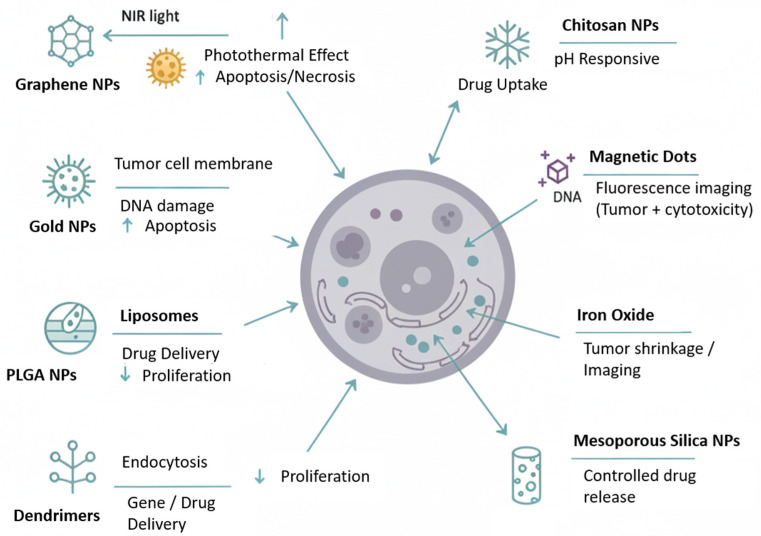
Summarize figure of nanoparticle-mediated tumor therapy mechanisms. The central illustration shows a tumor cell targeted by various classes of nanoparticles (NPs), highlighting their specific triggers, intracellular targets, and primary therapeutic outcomes. Graphene NPs utilize Near-Infrared (NIR) light for membrane ablation and apoptosis induction [103]. Gold NPs exert photothermal effects targeting the nucleus and mitochondria [104]. Liposomes and PLGA NPs facilitate sustained drug delivery and inhibit cell proliferation via the Enhanced Permeability and Retention (EPR) effect [105,106]. Dendrimers are shown delivering genetic material and drugs through endocytosis [107]. Chitosan and Mesoporous Silica NPs leverage pH responsiveness for controlled cargo release within lysosomes and the cytoplasm [108,109]. Iron Oxide NPs enable dual-mode tumor shrinkage and diagnostic imaging via magnetic field guidance [20,110]. Image generated with Gemini.

The high mortality rate of cancer is directly related to the failure of therapeutic responses, despite the continuous search for more effective compounds and the identification of new treatment targets [76,77,78,79]. In this context, nanoparticles represent another promising approach due to their unique properties, such as small size, high surface-to-volume ratio, good stability, and reactivity, making them suitable for cancer diagnosis, imaging, and therapy [80,81,82,83]. A variety of platforms are currently under investigation (Figure 6 and Figure 7), including metallic nanoparticles, which provide thermal stability and multifunctional capabilities for drug delivery and diagnostics [84,85,86]; lipid nanoparticles and liposomes, capable of encapsulating both hydrophilic and hydrophobic compounds and modifiable with targeting ligands [87,88,89]; polymeric nanoparticles and micelles, allowing sustained drug release, reduced toxicity, and demonstrated antitumor effects in early clinical trials [90,91,92,93]; and carbon nanotubes, which can stimulate immune responses by facilitating antigen and adjuvant delivery and promoting CD4^+^ and CD8^+^ T cell activation, thereby contributing to tumor inhibition [94,95].

Some of the strategies mentioned above have already been approved by the FDA, and others remain in preclinical development. FDA-approved examples include Doxil^®^ (liposomal doxorubicin) for breast cancer, Abraxane^®^ (albumin-bound paclitaxel) for pancreatic cancer, and Onivyde^®^ (liposomal irinotecan) for metastatic pancreatic adenocarcinoma in combination therapy [38]. Collectively, these advances illustrate the growing integration of nanotechnology, cancer biology, and materials chemistry, establishing nanomedicine as a central tool in oncology and opening new avenues for personalized and more effective therapies.

Among the diverse nanoparticle platforms, graphene-based materials and dendrimers have attracted particular attention due to their unique combination of physicochemical and biological properties. Graphene oxide (GOX), in particular, offers improved dispersibility, abundant oxygen-containing groups for functionalization, and a favorable balance between reactivity and biocompatibility. These features enable the construction of hybrid nanostructures, such as GOX-dendrimer systems, capable of interacting with multiple cellular pathways, potentially addressing several hallmarks of cancer simultaneously [96,97,98,99,100,101,102].

## 4. Modulating the Cellular Landscape: The Role of Graphene-Based Materials

**Figure 8:**
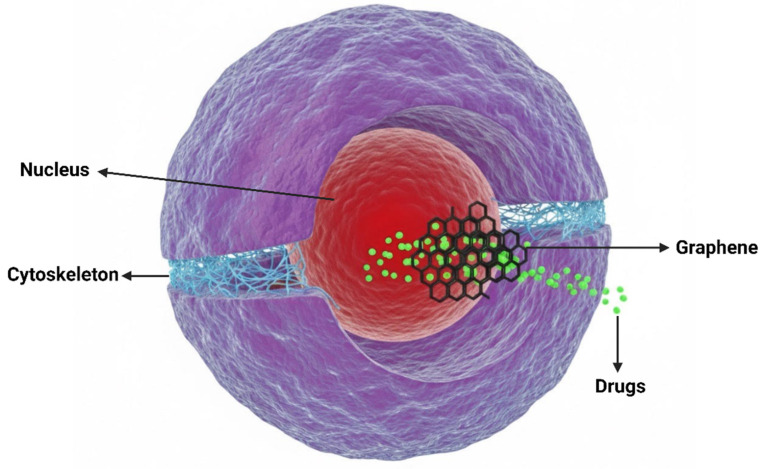
Biomedical applications for graphene-based materials as drug delivery systems. Image created by Gemini and modified with BioRender.

Strategies using graphene-based materials also represent a promising approach to cancer treatment. Graphene, isolated by Geim and Novoselov (2007), is one of the most studied crystalline forms of carbon due to its large surface area, high thermal stability, and remarkable mechanical strength [111,112]. Its two-dimensional hexagonal lattice structure provides properties suitable for applications in cancer treatment and diagnostics [113,114] (Figure 8). Despite its biomedical potential, a comprehensive understanding of the material’s interaction with different cell types is necessary, although low concentrations show minimal toxicity in mammalian cells [115,116,117,118,119].

It is important to note that graphene does not have a standardized morphology or size, which means different studies use materials with distinct characteristics, complicating comparisons [120,121,122]. Additionally, both graphene and its derivatives can interact with various cellular components, primarily affecting the plasma membrane, the cytoskeleton, and membrane-bound organelles such as the nucleus, mitochondria, and lysosome [123,124,125,126,127,128].

Graphene nanoparticles can be internalized by cells via multiple pathways, including clathrin-mediated endocytosis, caveolae-mediated uptake, endocytosis mediated by bio corona components, macropinocytosis, and can penetrate the plasma membrane directly. The internalization route is influenced by nanoparticle size, shape, surface charge, and functionalization [129,130]. Once internalized, graphene oxide (GOX) localizes in lysosomes, mitochondria, and cytosol, potentially compromising lysosomal integrity and altering mitochondrial function [131,132]. These interactions can trigger ROS production, autophagy, and apoptosis [133,134]. Beyond these effects, several studies indicate that graphene-based nanomaterials also reshape cellular metabolic processes. GOX exposure has been shown to impair enzymes related to drug-metabolism and alter lipid metabolic pathways, reflecting a broader metabolic reprogramming triggered by nanoparticle-cell interactions. In hepatocytes, sublethal GOX concentrations suppress cytochrome P450 activity and induce acute-phase responses. In contrast, in vascular smooth muscle cells GOX modulates antiviral signaling and lipid metabolism via TLR3 suppression [135,136]. Together, these findings demonstrate that GOX not only perturbs organelle function but also exerts systemic metabolic effects that influence immune and stress responses.

Within the range of biomedical applications of graphene-based materials, targeted drug delivery has emerged as a particularly promising strategy. This potential is attributed to graphene’s high surface area, versatile surface chemistry, and strong affinity for biomolecules, which collectively enable efficient drug loading and transport. Graphene quantum dots (GQDs), in particular, have been shown to enhance cellular uptake and intracellular release of chemotherapeutic agents, resulting in increased cytotoxicity in tumor cells and reduced off-target effects in healthy tissues. More recently, stimuli-responsive nanoplatforms have been developed to achieve controlled drug release in response to pH, glutathione levels, or near-infrared (NIR) irradiation, allowing the coordinated combination of chemotherapy and photothermal therapy. By integrating multiple therapeutic functions within a single nanoplatform, graphene-based systems can overcome limitations associated with drug solubility, targeting efficiency, and resistance mechanisms, thereby improving antitumor efficacy and minimizing systemic toxicity [137,138,139,140,141].

Graphene-based nanocomposites have also been extensively explored for cancer imaging and theragnostic applications, benefiting from their unique optical, electrical, and thermal properties. These materials enable multimodal imaging approaches, including fluorescence, and magnetic resonance imaging, allowing non-invasive tumor visualization and real-time monitoring of therapeutic delivery. Apart from their diagnostic function, the intrinsic photothermal conversion capability allows the integration of imaging with therapeutic modalities, highlighting these platforms as key components of emerging theragnostic strategies focused on addressing the complexity of cancer through combined diagnosis and treatment [142].

Functionalization with polymers such as polyethylene glycol (PEG) improves the solubility, colloidal stability, and circulation time of graphene-based nanomaterials. Importantly, PEG architecture modulates how immune cells interact with these materials. In primary human monocytes, GOX functionalized with linear PEG showed higher internalization, whereas branched PEG reduced the number of nanoparticles taken up by the cells. Despite these differences in uptake, GOX-PEG did not affect monocyte viability but altered functional responses, including a concentration-dependent reduction in phagocytosis, decreased detectable ROS levels, and selective stimulation of IL-10 secretion [143]. Besides these immunological effects, PEGylated GOX also influences cellular metabolism. In THP-1 cells, PEG-GOX nanoparticles modulated apoptosis, suppressed ROS production, altered cytokine secretion, and reprogrammed metabolic pathways. Larger PEG-GOX formulations suppressed oxidative phosphorylation and glycolysis within 24 h, demonstrating that even non-cytotoxic concentrations can profoundly reshape immune cell metabolic activity [144]. These findings reinforce that PEG surface architecture not only affects uptake and biocompatibility but also drives metabolic and functional reprogramming in immune cells. Recent studies have shown that graphene oxide-PEG nanoparticles effectively inhibit tumor growth in vivo, demonstrating the clinical potential of graphene-based PTT strategies [145]. Another application is in photodynamic therapy by conjugation with photosensitizing agents. This approach selectively induces cytotoxicity in tumor cells and can enhance the efficacy of conventional chemotherapeutics in combinatorial therapies [146,147,148].

Graphene-based nanocarriers can also be used for gene therapy, facilitating intracellular delivery of siRNA and miRNA to silence oncogenes or modulate tumor suppressor pathways. These platforms improve gene stability, cellular uptake, and endosomal escape, representing a promising approach to overcome chemoresistance in cancer cells. Functionalized graphene materials have been explored for immunomodulatory applications, including carriers for tumor-associated antigens or cytokines to enhance antitumor immune responses. Such platforms improve T cell activation and natural killer cell-mediated cytotoxicity, integrating immunotherapy with nanomedicine strategies [149,150,151].

Among graphene derivatives, graphene oxide (GOX) has received particular attention due to its abundant functional groups, high dispersibility in aqueous solutions, and ability to conjugate drugs and targeting moieties. GOX-based platforms interact with cellular membranes, the cytoskeleton, and organelles such as mitochondria and lysosomes, enabling both therapeutic action and intracellular delivery of bioactive compounds. Functionalization with polymers, peptides, or antibodies further expands GOX versatility [152,153,154,155,156,157,158].

## 5. Dendrimers: The Power of Branching

**Figure 9:**
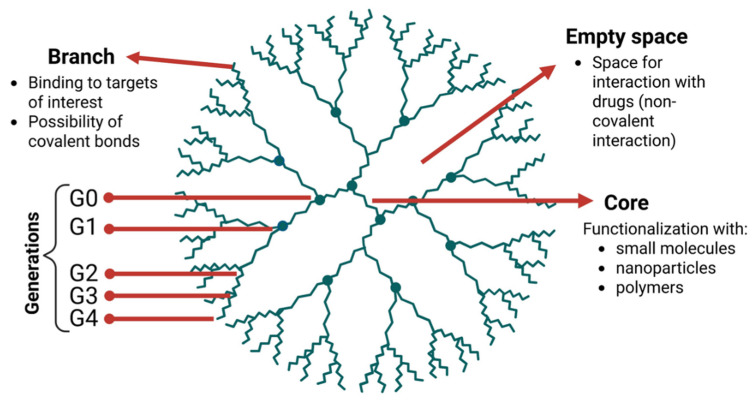
Schematic structural of a dendrimer. Image created with BioRender.

Dendrimers are highly branched and symmetrical macromolecules that resemble a tree with branches. They have a three-dimensional structure composed of: I) a core, II) successive layers of branched units (called ‘generations’), and III) terminal functional groups on the surface (Figure 9). They can be applied in drug delivery through two strategies: formulation, where the drug is physically encapsulated via non-covalent interactions, and nanoconstruction, where the drug is covalently bound to the structure. Polyamidoamine (PAMAM) and polypropyleneimine (DAB-AM-16) dendrimers are the most studied, as when conjugated with chemotherapeutic agents such as doxorubicin and cisplatin, they demonstrate improved therapeutic response compared to the free drug molecule [157,158,159,160,161,162].

Dendrimers are internalized predominantly through endocytic pathways, with uptake efficiency strongly influenced by generation number, size, and surface charge. Cationic PAMAM dendrimers interact electrostatically with negatively charged cell membranes, which enhances internalization but also increases the chances of membrane destabilization and cytotoxicity [163].

Studies in cancer cell models provide concrete evidence that dendrimers can contribute to therapeutic outcomes beyond acting as passive carriers, particularly by enabling efficient intracellular delivery of bioactive cargos. PAMAM-calix dendrimers form stable, positively charged complexes with regulatory siRNAs, efficiently internalize into cancer cells, and partially suppress cellular activity without inducing significant cytotoxicity, highlighting their potential as non-viral gene delivery systems for cancer nanomedicine [164]. Besides nucleic acid delivery, dendrimers have been widely explored as nanocarriers for poorly soluble natural compounds, owing to their controlled architecture, low polydispersity, and high functional group density, which collectively enhance drug solubility and bioavailability [165]. Targeting strategies further improve selectivity: trastuzumab-functionalized PAMAM dendrimers enhanced cellular uptake and antiproliferative activity in HER2-positive breast cancer cells (MDA-MB-453) compared to HER2-negative cells (MDA-MB-231). When conjugated with docetaxel, these targeted dendrimers promoted increased apoptosis and improved pharmacokinetic profiles, supporting their potential for selective delivery in HER2-positive breast cancer therapy [166]. Additionally, PAMAM–5-fluorouracil dendrimer complexes significantly reduced cell viability across multiple human cancer cell lines, including melanoma (A375), glioblastoma (SNB-19), prostate cancer (DU-145), and colon adenocarcinoma (HT-29), without inducing detectable toxicity in normal mouse fibroblasts, supporting their potential as in vitro anticancer drug delivery systems [167]. These findings highlight that dendrimers function not only as carriers but as versatile platforms capable of enhancing therapeutic efficacy and overcoming resistance mechanisms.

Functionalizing graphene oxide (GOX) with dendrimers has emerged as a promising strategy to create hybrid nanomaterials for drug delivery and theragnostic. These GOX-dendrimer hybrids combine the large surface area and stimuli-responsive properties of GOX with the branched functional groups of dendrimers, enabling: enhanced drug loading and stability, tunable surface chemistry for selective targeting, efficient cellular uptake through multiple endocytic pathways, and controlled intracellular release of drugs or genetic material [168,169,170].

## 6. Beyond Individual Components: Emergent Properties and Complementarity in Graphene-Dendrimer Hybrids

The development of graphene-dendrimer hybrids represents a sophisticated approach to material engineering (Table 2). When considered individually, graphene-based materials and dendrimers offer distinct but incomplete solutions for cancer nanomedicine. As mentioned above, graphene-based materials provide exceptionally high surface area, efficient drug loading through π-π stacking, and intrinsic photothermal/photodynamic properties. However, they often present limited control over surface chemistry, poor colloidal stability, and nonspecific biological interactions. Dendrimers present a highly defined three-dimensional architecture with precise control over size, surface charge, and ligand density, which enables tunable biological interactions and active targeting, whereas their drug loading capacity and stimuli-responsiveness are comparatively limited. Hybrid graphene-dendrimer systems overcome these individual limitations by integrating the structural and functional advantages of both components, which confer emergent properties. At the molecular level, dendrimer functionalization of graphene oxide improves colloidal stability and reduces aggregation through steric and electrostatic effects, simultaneously enabling multivalent interfaces for drug conjugation or receptor-specific targeting. In contrast, the graphene oxide scaffold contributes high drug-loading capacity, stimuli-responsive behavior, and photothermal/photodynamic functionalities, resulting in integrated theranostic platforms [159,160,161].

PAMAM-GOX nanocarriers exhibited increased drug-loading capacity and pH-responsive release, which enhanced cytotoxicity toward MDA-MB-231 breast cancer cells and maintained biocompatibility with non-tumorigenic lines (HEK 293T) [171]. Additional architectures, such as PEG-branched GOX platforms capable of co-delivering Pt(IV) prodrugs and doxorubicin, demonstrate synergistic antitumor activity and improved therapeutic indices [172]. Real-time microscopy studies further show that dendrimer-decorated GOX (PAMAM, DAB-AM-16) were internalized more efficiently in MCF-7 cells than unmodified GOX, supporting enhanced cellular uptake as a key benefit of dendrimer functionalization [116]. Collectively, these findings indicate that dendrimer-GOX hybrids improve drug loading, responsiveness to tumor microenvironment stimuli, and intracellular delivery, reinforcing their value in targeted cancer nanomedicine.

Despite their promise, these hybrid materials also present challenges and limitations. Cationic dendrimers can exacerbate cytotoxicity and oxidative stress when combined with GOX, particularly at higher generations, due to strong electrostatic interactions that destabilize cellular membranes and organelles [173]. Additional concerns include complex synthesis and variability due to the integration of two distinct nanomaterials, as well as the potential for off-target accumulation in organs such as the liver and spleen when targeting is not adequately optimized [174,175].

In conclusion, the obtention of GOX-dendrimer hybrids requires precise control over multiple parameters, including dendrimer generation, degree of functionalization, GOX sheet size, and conjugation chemistry. These factors critically influence the reproducibility of hybrid structures, uniformity of drug loading, and predictability of cellular interactions. Fabrication remains non-trivial, demanding careful optimization to balance therapeutic performance, biocompatibility, and manufacturability.

## 7. The Nano-Bio Interface: Deciphering the Cellular Impact of GOX and Dendrimers

The interaction of nanomaterials with cells and tissues is critically influenced by their biological identity, which is largely determined by the formation of a protein corona upon exposure to biological fluids. The protein corona is a layer of proteins and biomolecules on nanomaterials that shapes their biological identity and influences how cells recognize and absorb them. This dynamic layer of adsorbed biomolecules modulates recognition, internalization, and intracellular fate, thereby affecting uptake efficiency, intracellular trafficking, cytotoxicity, and activation of stress-related signaling pathways. The composition of the protein corona varies between in vitro and in vivo conditions, reflecting the source of biomolecules, and can confer new biological properties that profoundly influence cellular responses [176,177,178,179,180].

**Figure 10:**
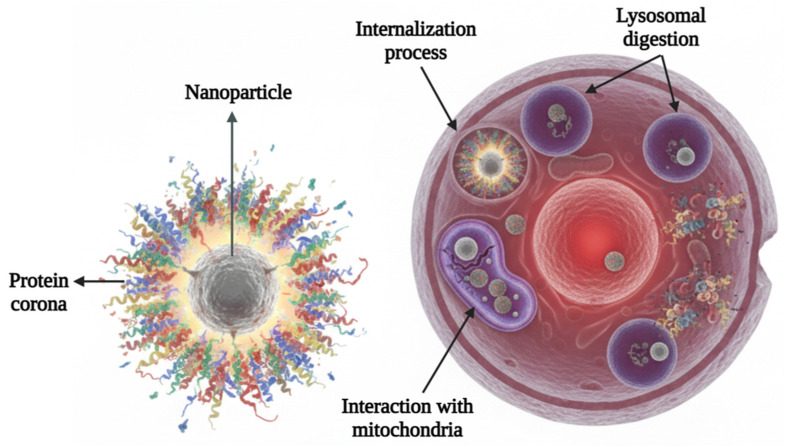
Schematic representation of the cellular endocytosis and intracellular processing of nanomaterials, illustrating general uptake pathways and lysosomal digestion. Image created in Gemini and modified with BioRender.

Following cellular internalization, nanomaterials can be sequestered within endo-lysosomal compartments or escape into the cytosol, where they may interact with a range of intracellular organelles, including mitochondria, the endoplasmic reticulum, and the nucleus (Figure 10) [181]. These interactions are highly dependent on nanomaterial physicochemical properties such as size, shape, surface charge, and rigidity, which collectively determine the route of uptake and intracellular distribution. Importantly, these post-internalization events are not biologically neutral and can induce major cellular stress responses and cell fate-related pathways [182,183,184].

As we mentioned before, cationic dendrimers exhibit high affinity for negatively charged cell membranes, promoting efficient internalization but also increasing the possibility of cell damage [185,186]. Once internalized, these positively charged dendrimers can disrupt mitochondrial homeostasis, leading to loss of mitochondrial membrane potential, elevated ROS production, and activation of intrinsic apoptotic pathways. Such mitochondrial and oxidative perturbations can further interfere with cell-cycle regulatory mechanisms, promoting G0/G1 arrest or apoptosis depending on surface chemistry and functionalization [187]. Together, these findings demonstrate that dendrimer charge and architecture critically shape cellular responses, influencing key cancer hallmarks such as resisting cell death, sustaining proliferative signaling, and genomic instability [188,189].

Graphene oxide, as a two-dimensional nanomaterial, displays distinct cellular interaction mechanisms compared to spherical nanoparticles. Its sheet-like morphology enables strong physical interactions with the plasma membrane, including loss of membrane integrity, alterations in mechanical properties, and local disruption, which can facilitate cellular uptake or trigger stress responses [126,190]. Once internalized, GOX accumulates in lysosomes, where it may compromise lysosomal membrane integrity and modulate autophagic flux. Notably, this property can be therapeutically used, as the acidic lysosomal environment enables the pH-responsive release of anticancer agents from graphene-based materials, facilitating intracellular drug release following lysosomal uptake [131]. GOX exposure has been consistently associated with elevated ROS production, mitochondrial damage, cytoskeletal remodeling, and activation of autophagy- and apoptosis-related pathways [191,192].

Dolgikh et al. showed that PEGylated graphene oxide nanoparticles were efficiently internalized by MCF-7 breast cancer cells and could function as photothermal agents under near-infrared (NIR) irradiation, exhibiting increased apoptosis and cell death following localized hyperthermia, although without statistically significant cytotoxicity under the tested conditions [193]. Subsequent work demonstrated that reduced graphene oxide showed a more pronounced pro-apoptotic effect in MCF-7 cells by inducing mitochondrial dysfunction, reactive oxygen species (ROS) generation, loss of mitochondrial membrane potential, and activation of NF-κB-associated signaling, ultimately leading to Bax/Bcl-2–mediated programmed cell death [194]. Further supporting the critical role of surface chemistry, functionalized graphene-based nanocomposites, such as highly reduced graphene oxide-gold systems, displayed enhanced dispersibility and cellular interaction, resulting in significantly amplified apoptosis induction in breast cancer cells compared with pristine or nonfunctionalized graphene derivatives [195]. Collectively, these studies demonstrated that both physicochemical properties and surface functionalization critically governed how graphene-based nanomaterials disrupted cellular homeostasis and activated apoptosis-related pathways in cancer cells.

In addition to isolated cellular effects, perturbations induced by nanomaterials on intracellular signaling and organelle function provide important insight into how cancer cells adapt to environmental stress. For example, oxidative stress and mitochondrial dysfunction triggered by dendrimers, graphene oxide (GOX), or hybrid GOX-dendrimer materials can activate compensatory survival pathways, reflecting mechanisms exploited by tumor cells during disease progression and therapy resistance [196,197]. Similarly, alterations in cytoskeletal organization and membrane dynamics can affect migratory behavior and invasive potential, offering experimental parallels to the hallmark of activating invasion and metastasis. By modulating redox balance, intracellular trafficking, and cell cycle regulation, these nanomaterials reveal mechanisms of tumor adaptation to environmental stress, and simultaneously provide versatile platforms for drug delivery and theragnostic applications [198,199,200].

Overall, the cellular responses to these nanomaterials extend beyond conventional toxicity assessments, acting as informative probes to elucidate the molecular and cellular bases of cancer hallmarks. Careful design and characterization of these hybrids are essential to balance therapeutic efficacy, safety, and manufacturability, highlighting their translational potential at the interface of nanomaterial engineering and cancer cell biology.

## 8. Conclusions

Cancer remains a major therapeutic challenge, and graphene-based nanomaterials, particularly when integrated with dendritic polymers, offer a powerful framework for the rational design of next-generation anticancer strategies. Importantly, discussing graphene oxide and dendrimers within a unified conceptual framework is scientifically meaningful, as these materials address complementary dimensions of nano-bio interactions, such as surface chemistry, cellular uptake, intracellular trafficking, and stimulus-responsive behavior.

By linking graphene-cell interactions to the Hallmarks of Cancer, this review moves beyond a materials-centered description. Instead, it provides a mechanistic perspective that connects nanomaterial design principles to tumor adaptation, therapeutic response, and translational potential. Graphene oxide and dendrimers each exhibit intrinsic functional advantages, and their hybridization creates a coupled system in which interfacial interactions reshape the functional behavior of both components. The combination leads to enhanced colloidal stability, high drug-loading capacity, multivalent and cooperative cellular interactions, and integrated stimuli-responsive therapeutic functions.

These emergent properties significantly reshape how cancer cells sense, internalize, and respond to nanomaterials, enabling levels of control that are not achievable with either component alone under comparable conditions. Consequently, hybrid graphene-dendrimer platforms should be considered as a distinct design paradigm in cancer nanomedicine. Finally, a comprehensive understanding of cellular responses to both pristine and hybrid nanomaterials is essential to guide the rational development of safe and efficacious nanotherapeutics, and to accelerate their clinical translation.

## 9. Future Perspective

The combined evidence discussed in this review demonstrates that graphene-based nanomaterials and dendrimers are not biologically inert platforms, but active modulators of cellular behavior. Through their interactions with cellular membranes, organelles, and intracellular signaling networks, these nanomaterials can directly influence processes that are central to cancer progression. Consequently, future research should move beyond their conventional use as passive delivery vehicles and instead focus on their capacity to selectively interfere with cancer-associated cellular vulnerabilities and hallmark-related pathways [201,202].

A critical priority for future studies is the establishment of clearer structure-activity interactions linking nanomaterial physicochemical properties, such as size, morphology and surface charge, to specific cellular responses. Key parameters, including protein corona composition, uptake mechanisms, intracellular trafficking, and organelle targeting, should be systematically correlated with downstream biological outcomes, such as oxidative stress, mitochondrial dysfunction, cell cycle arrest, and cell death. Such mechanistic insight is essential to improve predictability, reduce off-target toxicity, and enable rational comparison between nanomaterials with fundamentally different geometries, including two-dimensional graphene oxide sheets and three-dimensional dendritic structures [203,204,205].

Importantly, many of the cellular effects induced by these nanomaterials intersect directly with multiple hallmarks of cancer. Nanomaterial-induced oxidative stress and mitochondrial impairment challenge the hallmark of resisting cell death, while alterations in cell cycle dynamics and proliferative signaling may selectively affect tumor cells division. In parallel, membrane perturbation and cytoskeletal remodeling caused by nanomaterial interactions may influence invasion-related processes and metastatic potential. Understanding the interactions between nanomaterials and cells in the context of cancer, therefore, provides a unifying conceptual idea to guide the development of mechanism-based anticancer nanotechnologies [206].

From a translational perspective, future work should prioritize biologically relevant experimental models. Advanced three-dimensional cultures, tumor organoids, and microfluidic systems better recapitulate the tumor microenvironment and are expected to provide more predictive data regarding nanomaterial distribution, efficacy, and toxicity. In addition, long-term and low-dose exposure studies will also be crucial to understand chronic cellular adaptations, particularly for sustained administration [207,208,209].

Finally, the development of multifunctional graphene-based platforms integrating imaging, targeted delivery, and therapeutic functions holds significant promise for precision oncology. However, clinical translation will depend on standardized synthesis protocols, rigorous physicochemical characterization, and reproducible biological evaluation. By integrating materials science with cancer biology and clinically relevant models, future research can shift the field from proof-of-concept studies toward robust, mechanism-informed nanomedicine approaches capable of addressing the complexity of cancer [210].

## Figures and Tables

**Table 1:** Representative nanomaterials used in cancer research and therapy, their main applications, clinical status, and associated challenges.

Cancer Type/Model	Nanomaterial/ Derivative	Applications	Clinical Status	General Concerns
Multiply solid tumor (breast cancer, glioblastoma, melanoma)	Graphene Oxide (GOX)	Drug delivery, photothermal/photodynamic therapy; apoptosis induction	Preclinical (in vitro/in vivo studies)	Biocompatibility, toxicity and systemic elimination
Solid tumors (head and neck, lung, glioblastoma)	Gold Nanoparticles (AuNPs)	Photothermal therapy, radiosensitization, drug delivery, imaging	Clinical trials ongoing (TNF-α AuNPs Phase 1, Trial NCT03020017 studied spherical gold NPs)	Long-term safety, accumulation, immune responses
NSCLC adenocarcinoma	Liposomal Cisplatin (Lipoplatin)	Targeted cisplatin delivery with reduced toxicity	Completed Phase I–III trials in cancer with superior efficacy to conventional cisplatin in combination therapy	Regulatory approval pending in many regions
Various solid tumors	Lipid Nanoparticles (LNPs)	Nucleic acid delivery (siRNA/miRNA), chemotherapy carriers	Used clinically, including in FDA-approved medications (liposomal irinotecan for metastatic pancreatic adenocarcinoma) LNPs carrying siRNA/miRNA/nucleotides are in clinical trials for solid tumors (INT-1B3 targeting miR-193a-3p in solid tumors). Other LNPs in Phase 1/2 against solid tumors and various types of cancer	Off-target effects, immune recognition
Advanced solid tumors	PLGA Polymeric NPs	Controlled release of chemo and immunotherapy agents	Preclinical and early clinical (not FDA-approved as monotherapy) (PRECIOUS-01 with NY-ESO-1 antigen in solid tumor)	Scale-up, reproducibility, targeting efficiency
Glioblastoma and leukemias	Dendrimers (e.g., PAMAM)	Drug/gene delivery, targeting ligands	Preclinical exploration; no approved products yet	Potential toxicity, clearance issues
Multiple cancers	Iron Oxide NPs	MRI contrast, hyperthermia, immune activation, targeted delivery	NanoTherm (iron oxide) is in Phase 2b for prostate cancer Feraheme^®^ (coated iron oxide) is electronically approved by the FDA as an imaging agent, with applications explored in breast/triple-negative, head and neck, and lung cancer imaging assays	Accumulation, long-term effects
Ovarian, metastatic breast, Kaposi’s sarcoma	Liposomal Doxorubicin (Doxil/Caelyx)	Approved chemotherapeutic NP	FDA-approved	Cardiotoxicity reduced vs. free drug
Kaposi’s sarcoma	Liposomal Daunorubicin (DaunoXome)	Chemotherapy delivery	FDA-approved	Cardiotoxicity reduced vs. free drug
Acute myeloid leukemia	Liposomal Cytarabine + Daunorubicin (VYXEOS/CPX-351)	Combination chemotherapy	FDA-approved	Classic chemo side-effects

Note: Data from these studies [9,10,11,12,13,14,15,16,17,18,19,20,21,22,23,24,25].

**Table 2:** Comparative summary of graphene, dendrimers, and graphene-dendrimer hybrid systems illustrating complementary functionalities and emergent properties relevant to cancer nanomedicine [152,153,154,155,156,157,158,159,160,161,162,163,164,165,166,167,168,169,170,171,172,173,174,175].

Properties	Graphene-Based Materials	Dendrimers	Graphene-Dendrimer Hybrids
Structural organization	Two-dimensional sheets	Three-dimensional, highly branched architecture	Hierarchical 2D-3D hybrid structure
Surface area	Very high	Moderate	High and accessible
Drug loading capacity	High (π-π stacking, adsorption)	Limited to moderate	High
Control over surface chemistry	Limited precision	Highly precise and programmable	Multiscale and controllable
Colloidal stability	Often poor due to aggregation	Generally good	Improved via steric and electrostatic stabilization
Targeting capability	Predominantly passive	Active (ligand conjugation)	Passive + multivalent active targeting
Cellular uptake	Variable and cell-type dependent	Efficient but charge-dependent	Enhanced internalization efficiency
Emergent properties	Intrinsic 2D-related photothermal effects, redox- and pH-responsive behavior	Architecture-driven multivalency, charge-dependent cellular interactions	Coupled stimuli-responsiveness, synergistic uptake, multifunctionality
Main limitations	Aggregation, nonspecific interactions	Cytotoxicity at high generations	Synthetic complexity, optimization-dependent toxicity

## Data Availability

No new data were created or analyzed in this study. Data sharing is not applicable to this article.
